# Interaction between Parental Education and Household Wealth on Children’s Obesity Risk

**DOI:** 10.3390/ijerph15081754

**Published:** 2018-08-15

**Authors:** Yang Liu, Yanan Ma, Nan Jiang, Shenzhi Song, Qian Fan, Deliang Wen

**Affiliations:** Institute of Health Science, China Medical University, No. 77 Puhe Road, Shenyang North New Area, Shenyang 110122, China; yliu0568@cmu.edu.cn (Y.L.); ynma@cmu.edu.cn (Y.M.); ndjiang@live.ca (N.J.); song_shenzhi@163.com (S.S.); 18040209895@163.com (Q.F.)

**Keywords:** childhood obesity, parents’ education, household wealth, health inequalities

## Abstract

Parents’ education and household wealth cannot be presumed to operate independently of each other. However, in traditional studies on the impact of social inequality on obesity, education and financial wealth tend to be viewed as separable processes. The present study examines the interaction of parents’ education and household wealth in relation to childhood obesity. Anthropometric measurement and questionnaire surveys were carried out on 3670 children (aged 9–12 years) and their parents from 26 elementary schools in northeast China. Results showed that the interaction term was significant for household wealth and father’s education (*p* < 0.01), while no significant interaction between household wealth and mother’s education was found. In a separate analysis, the interaction was statistically significant among girls for obesity risk based on BMI (*p* = 0.02), and among urban children for both obesity risk based on BMI (*p* = 0.01) and abdominal obesity risk based on WHR (*p* = 0.03). Specifically, when household wealth increased from the first quintile to the fifth quintile, OR for father’s education decreased from higher than 1 (OR = 1.95; 95% CI: 1.12–3.38) to non-significant for girl’s obesity risk, from non-significant to lower than 1 for urban children’s obesity risk (OR = 0.52; 95% CI: 0.32–0.86 for the fourth quintile; OR = 0.37; 95% CI: 0.19–0.73 for the fifth quintile) and from higher than 1 (OR = 1.61; 95% CI: 1.04–2.05) to non-significant for urban children’s abdominal obesity risk. These findings indicate that father’s education level interacts with household wealth to influence obesity among girls and urban children in northeast China.

## 1. Introduction

In the last 40 years, obesity has become one of the most serious nutritional concerns for children and adolescents, affecting countries rich and poor, with the global number of obese children and adolescents rising more than tenfold, from 11 million in 1975 to 124 million in 2016 [1,2]. The obesity epidemic in younger age groups brings about a large increase in the incidence of various morbidities and shortened life expectancy [3]. Socioeconomic status (SES) and economic insecurity were hypothesized to be one of the key determinants of obesity prevalence and other chronic diseases [4]. Tackling social distribution of obesity risk in early life is a main challenge in childhood obesity prevention and has also been recommended as an effort to tackle inequalities in mean BMI and obesity status across all ages [5].

Household wealth and parents’ education were the most reported economic and social dimensions of SES that could influence children’s obesity risk [6,7,8]. Household wealth influences the material environment to which children are exposed. Low income may be related to food access dilemmas resulting from resource constraints and adverse food environments [9,10]. Parent education levels affect parents’ ability to process health information, which leads to improved health-related decisions in parenting practice and which also affects their motivation to adopt a healthy lifestyle as role models for their children [11,12]. Children of more educated parents were reported to be more likely to eat breakfast and consume fewer calories from snacks and sweetened beverages [9].

The majority of previous studies viewed the impact of income and education on obesity risk as separable processes. However, education and income cannot be presumed to operate independently of each other considering their high correlation [13,14]. Additionally, both income and education have been reported to relate to obesity in nonlinear ways [7,15,16]. With income and education levels varying, the social distribution of obesity risk also varies. This necessitates research on how social inequalities may be more important in different settings and the importance of different social factors on different people groups [13].

According to the nutrition transition model [17], countries follow an economic and nutritional progression from Stage 1 of collecting food and Stage 2 of famine to Stage 3 of receding famine, followed closely by Stage 4 of nutrition-related non-communicable disease, and finally Stage 5 of the development or introduction of behavioral change, including diet modification and increased recreational activity. China is a classic example of a country moving towards nutrition transition to Stage 4, illustrating nutrition transition in the developing world [18]. Accompanied by the nation’s rapid economic growth and significant social and cultural change within the last three decades, the prevalence of overweight and obese children in China increased by 35.22 (boys) and 25.21 (girls) percentage points (from 1.91% for boys and 1.53% for girls in 1985 to 37.13% for boys and 26.74% for girls in 2014) based on the International Obesity Task Force criteria for children aged 7–12 years [19]. Learning from the tremendous change in China may help with designing nutrition policies and interventions to reduce health hazards during the nutrition transition.

Therefore, this study explored the social distribution of childhood obesity risk in China and investigated the interaction between parents’ education and household wealth on children’s obesity risk. We hypothesize in China’s family sets that higher education in parents would be negatively correlated with obesity in their children, but this higher education in parents would not have a significant role until household wealth reached a higher level.

## 2. Methods

### 2.1. Study Populations and Sampling

From 17 May to 23 June 2017, data was collected from fourth-, fifth-, and sixth-grade students and their parents from 26 elementary schools in Shenyang, China. This age range was sampled due to it being a time of striking behavioral change related to the social environment. Shenyang is the largest city in northeast China by urban population and consists of 13 administrative districts, including 10municipal districts of Shenyang proper, 1 count-level city, and 2 counties. According to the 2010 census, Shenyang’s total population had surpassed 8.1 million, with the urban population comprising 6.3 million of the total.

Two schools from each of the 13 administrative districts of Shenyang city were randomly selected, from which one class from each of the fourth-, fifth-, and sixth-grade divisions of each school was selected to be included in the study. All students from the selected classes and their parents were included as the sample of the present study with their consent, and all participants had the option to withdraw from the study at any point.

### 2.2. Data Collection

Anthropometric measurement—including height, weight, waist circumference, and hip circumference—was carried out on physical examination day at each school by trained investigators using techniques prescribed by Lohman et al. [20]. Household questionnaires on family demographics and each household’s wealth index were handed out to each student three days before physical examination, answered by one or both parents, and collected at physical examination. Incomplete questionnaires or those with missing data were filtered by the school teachers, school physician, or research personnel (SZS) and returned to parents for completion of any inadvertently missed portions. Written informed consents were obtained from all participating households before anthropometric measurement. The study was approved by the China Medical University Ethics Committee (71774173).

### 2.3. Measurements and Variable Definitions

Weight was measured using a portable Tanita DC-430MA dual frequency body composition monitor (TANITA Corporation, Tokyo, Japan). Standing height was measured without shoes, by a Seca 213 portable stadiometer (Hamburg, Germany). BMI was calculated as weight (kg) divided by height (m) squared. Waist circumference (WC) (cm) was measured at the midpoint between the level of the xiphoid process and the top of the iliac crest, and hip circumference (HC) (cm) at the widest point around the buttocks. Waist–hip ratio (WHR) was calculated as WC divided by HC. Obesity was defined according to cut-off BMI recommended by Working Group on Obesity in China (WGOC), as P95, based on data collection by the Chinese National Survey on Students Constitution and Health on primary and secondary school students ages 7 through 18 (Table 1) [21]. Abdominal obesity was defined by previously published WHR references based on Chinese children and adolescents living in Beijing (WHR ≥ P97) (Table 2) [22].

In China, all citizens must attend school for at least nine years, known as the nine-year compulsory education, which is free for citizens and is funded by the government. The compulsory education includes six years of primary education, starting at age six or seven, and three years of junior secondary education (junior middle school) for ages 12 to 15. Beyond compulsory education, Chinese citizens could choose to pursue higher education via qualification examinations at each stage, which could include three years of senior middle school education, any additional vocational training or apprenticeship, and any university education in a post-secondary degree program. Tuition for higher education is no longer subsidized by the government. Hence, in the present study, parental education consisting of the father’s education and the mother’s education and was divided into two levels: (1) no or compulsory basic education and (2) higher education.

The household wealth index was based on the following indicators: household income, food costs as a proportion of annual income, ratio of income to expenditure, self-reported evaluation of household income compared to the local average, income growth in the last three years, satisfaction of household income, number of private cars, number of computers, if the child has his/her own room, and number of family trips per year. The index was generated through a principal components analysis using the Filmer and Pritchett method to calculate factor loadings and derive a score for each household [14,23,24,25]. Household wealth levels were divided into quintiles: first = lower than or equal to 20% of the study sample, second = 21–40%, third = 41–60%, fourth = 61–80%, and fifth = higher than 80% of the study sample [24]. Sensitivity analysis was performed to classify the lowest 40% of households into “poor”, the highest 20% as “rich” and the rest as the “middle” group [24].

According to the residential registration system of China, citizens belonged to two residential groups: “urban” and “rural”. This data was collected as an item in the questionnaire.

### 2.4. Statistical Analysis

STATA 14.0 survey commands (svy) were used to analyze the data and account for the complex design effect, taking into account the effect of clustering and unequal weights as appropriate when computing frequencies, proportions, variance, standard errors, and confidence intervals.

Firstly, prevalence of obesity by subgroup was computed using a chi-squared test to compare the differences between sex, residence registration area, household wealth level, and parents’ education level.

Secondly, according to the hypothesis proposed by the present study, father’s education level and mother’s education level were focal independent variables, and household wealth was the moderator variable. Interaction terms between parents’ education level (no or compulsory basic education/higher education, reference category = no or compulsory basic education) and household wealth quintiles in continuous forms were fitted into logistic regression models. The interaction terms were examined for significance using the Wald test (the null hypothesis being that the interaction terms in the regression model were equal to zero) and the likelihood ratio (LR) test (examining whether the data was better fitted using a model with or without the interaction).

Next, considering the characteristics of obesity and Chinese society, analyses were performed separately for boys and for girls and then for urban residences and for rural residences. Firstly, boys and girls differ in body composition, patterns of weight gain, hormone biology, and susceptibility to certain social, ethnic, genetic, and environmental factors. In addition, social economic status was reported to influence boys’ and girls’ obesity statuses differently. Secondly, as a developing country, China is characterized by the urban–rural dual structure. Large disparities exist between urban and rural areas, including but not limited to the urban and rural income gap, different social security systems, and unequal distribution of public resources.

Finally, ORs and the confidence interval for the local independent variables (father’s education level and mother’s education level) were calculated at different values of the moderator by transforming the continuous moderator variable (household wealth quintile in its continuous form) and then rerunning the regression analysis.

All analyses were performed twice, first using obesity defined by BMI and then using abdominal obesity defined by WHR as dependent variables, in sequence. All analyses were carried out using the software STATA 14.0 (Stata Corp., College Station, TX, USA). Statistical significance was defined by a *p*-value of <0.05.

## 3. Results

The coverage attained by the anthropometric examination was 3670 (95% of the total number of enrolled students from selected grades 4, 5, and 6 of the 26 schools). As shown in Table 3, the average age of children in the present study was 10.8 ± 1.0 years. Forty-nine percent of the children were girls and 44.8% of the children were from urban families. The overall prevalence of obesity was 17.0%, defined by BMI, and the overall prevalence of abdominal obesity was 8.1%, defined by WHR. Table 3 also shows distribution of parents’ education in each household wealth quintile. As shown in Table 4, boys had a statistically higher obesity prevalence compared to girls (*p* < 0.001). There was no significant difference in childhood obesity prevalence between different household wealth levels, father’s education levels, mother’s education levels, or residence registration area. There was no significant difference in childhood abdominal obesity prevalence between different sexes, household wealth levels, father’s education levels or mother’s education levels or residence registration area.

As shown in Table 5, the interaction terms between household wealth and father’s education was significant for children’ obesity risk (*p* < 0.01 for interaction term). The LR test for whether the model was better fit with or without the interaction terms also provided strong evidence of the interaction effect of household wealth and father’s education on children’ obesity risk (*p* < 0.05).

Table 6 showed the separate analysis by sex. Interaction terms between household wealth and father’s education was significant for girls’ obesity risk (*p* < 0.05 for interaction terms) but not for that of boys.

Table 7 showed the separate analysis by residence area *(*urban/rural). The interaction terms between household wealth and father’s education were significant for children’s obesity risk (*p* < 0.05 for interaction term) and abdominal obesity risk (*p* < 0.05 for interaction term) in urban areas but not in rural areas.

**Figure 1:**
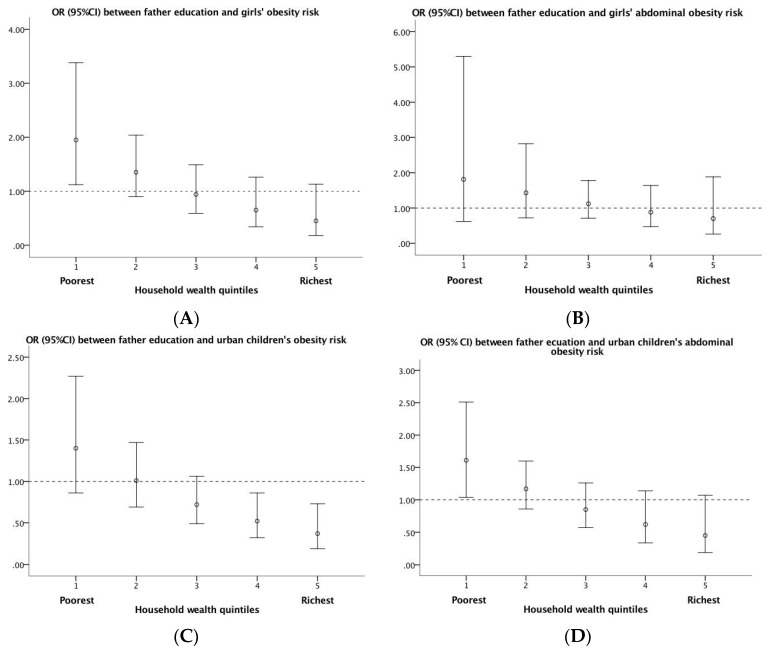
OR (95% CI) for father’s education level at different values of the household wealth quintile among girls and among urban children. (**A**) OR (95% CI) between father education and girls’ obesity risk; (**B**) OR (95% CI) between father education and girls’ abdominal obesity risk; (**C**) OR (95% CI) between father education and urban children’s obesity risk; (**D**) OR (95% CI) between father education and urban children’s abdominal obesity risk.

Figure 1 demonstrated details of the interaction between household wealth and father’s education on children’s obesity by showing OR (95% CI) for father’s education within different household wealth quintiles. When household wealth increased from the first quintile to the fifth quintile, OR for father’s education decreased from higher than 1 (OR = 1.95; 95% CI: 1.12–3.38) to non-significant for girl’s obesity risk (Figure 1A), from non-significant to lower than 1 for urban children’s obesity risk (OR = 0.52; 95% CI: 0.32–0.86 for the fourth quintile; OR = 0.37; 95% CI: 0.19–0.73 for the fifth quintile) (Figure 1C) and from higher than 1 (OR = 1.61; 95% CI: 1.04–2.05) to non-significant for urban children’s abdominal obesity risk (Figure 1D).

Results of sensitivity analysis (classifying the lowest 40% wealth index of households into “poor”, the highest 20% as “rich”, and the rest as the “middle” group) showed a similar interaction pattern between household wealth and parental education (Tables S1–S3, Figures S1 and S2).

## 4. Discussion

This study was one of the first to report on the interaction between parents’ education level and household wealth in childhood obesity risk. The interaction term was significant for household wealth and father’s education. No interaction between household wealth and mother’s education was found. In separate analysis, the interaction was statistically significant among girls for obesity risk based on BMI, and among urban children for both obesity risk based on BMI and abdominal obesity risk based on WHR.

### 4.1. Comparison with Prior Studies and Plausible Mechanisms

The present study examined how household wealth interacted with father’s education by showing the association between father’s education and children’s obesity risk in different household wealth quintiles. According to the results, when household wealth increased, OR for father’s education decreased from higher than 1 to non-significant for girl’s obesity risk, from non-significant to lower than 1 for urban children’s obesity risk, and from higher than 1 to non-significant for urban children’s abdominal obesity risk. Change of the association, which is from positive to non-significant or from non-significant to negative with the increase in household wealth, is partially consistent with our hypothesis that education would not be negatively associated with obesity until income reaches a certain level. The change may be explained by the “Obesity Kuznets curve” and nutrition transition [16,17,18]: as income rises, people consume more calories and obesity rates increase; as income continues to rise, personal health becomes a more valued asset to decrease obesity levels. During this process, education is believed to act as a “social vaccine” and bring the decrease in obesity level when income reaches a certain level [7]. A systematic review showed that the relationship between education and obesity was modified by the country’s economic development level and that an inverse association between educational attainment and obesity was more common in studies of higher-income countries and a positive association was more common in lower-income countries [7]. A previous ecological study also showed that culture development would not take effect to attenuate obesity prevalence until a country’s economy developed to a certain level [26].

The sex-dependent result where only girls are significantly affected by their fathers’ education level and household wealth is consistent with previous findings that indicate females are more vulnerable to socioeconomic conditions [27,28,29,30]. For example, obesity risk between different ethnic groups in the United States was greater in females [30]; and the relationship between education level and obesity was stronger among women, both in developed countries and in developing countries [16]. In addition, it was reported that girls are more influenced by parental practice than boys, and parental control was more significant among overweight or obese girls, which was not observed among boys of the same weight groups [27,31].

Socioeconomic status (SES) should not be explored without considering the macro social environment that individuals are exposed to [14,32]. In the present study, parents’ education level interacted with household wealth to correlate with obesity risk in urban children but not in rural children. There are several likely explanations. Firstly, rural areas tend to adhere to more traditional lifestyles. Parents with different education levels may not necessarily indicate very diverse ways in parenting practice. Secondly, food environment and built environment tend to have less diversity in rural areas. Different levels of household wealth may not be able to provide as diverse a material environment for children in rural areas as compared to in urban areas due to lack of options.

The disparity of parental roles on child health was reported by several previous studies [33,34,35]. The present study suggests that only higher education in fathers was linked with lower obesity risk when household wealth increased. One possible reason for this is that the education–overweight association is believed to be described by an inverted U-shaped curve [7,36]. As the nutrition transition progresses in a country, education acts as a “social vaccine” against increasing risk of overweight or obesity. In developing countries like China, paternal characteristics reflect the socioeconomic status of the entire household, and the father’s parenting often determines the extent of support from the family as a whole for the child’s weight loss and maintenance. For mothers, however, food provision and child nutrition practices were central to the traditional constructs of mothering [36], causing the downward turning point on the inverted U-shaped education-overweight curve to not be as apparent as that for fathers when mothers place more focus on feeding their children and less on reducing habits of overconsumption. Additionally, the effect of higher education levels of mothers on feeding practice is offset or reversed by a decrease in the time spent on childcare when higher education is often linked with more time-consuming and mentally demanding careers. Decreased attention from career-driven mothers may leave children vulnerable to obesogenic environments or to being overfed by grandparents [37,38]. Further qualitative studies are needed to delve deeper into the difference between the father’s role and the mother’s role in children’s obesity risk.

### 4.2. Implications for Intervention

The present study suggests, especially for countries undergoing nutrition transition Stages 1 through 4, that higher education in fathers may negatively relate to children’s obesity risk with increasing wealth. Whereas many current intervention programs target the matriarchs, further longitudinal evaluation of the effect of father’s education on children’s obesity risk may incorporate fathers into family intervention plans. In addition, we call for nutrition interventionists to take the family socioeconomic context into consideration when designing family-based interventions. The effects of nutritional interventions would not persist without considering the families’ socioeconomic context. In this way, nutrition and parenting based interventions could be more tailored to each family’s own unique situation.

The study boasts several strengths. Its main contribution lies in the interaction between parents’ education and household wealth on shaping the socioeconomic context of obesity risk, which was not previously explored among children. This went beyond simple uni-dimensional analyses of SES and childhood obesity and examined how they intersect. In addition, considering the disease feature of obesity and the social features of China, the present study went further to test the difference of the interaction between boys and girls and between urban areas and rural areas. The processes giving rise to illness and health are often inherently complex, especially for conditions such as obesity. This requires going beyond uni-dimensional analysis based on only one economic class, gender, caste, or ethnicity [13]. Different from traditional literature on the impact of social diversity on health, a fundamental hypothesis in the present study is that multiple dimensions interact with each other to shape the distribution of obesity. Secondly, the obesity status of children was measured by both BMI as obesity and by WHR as abdominal obesity, which were used to confirm the consistency of associations. Furthermore, household wealth was measured by a series of income-related questions and family affluence questions, which provided a more comprehensive outcome than simply studying household income as a single measure—as seen in most previous studies.

The present study is not without limitations. Firstly, this was a cross-sectional study where all co-variates were measured simultaneously, so causal inferences cannot be made. A longitudinal study is warranted to resolve the effect of father’s education and household wealth on children’s obesity risk. However, where this may be the main concern for studies among adults, the SES is measured by parents’ characteristics when studying children, and therefore reverse causality and health selection, would be rare [39]. Secondly, while investigating the interaction between parental education and household wealth, sex of children and residence area were also taken into account and presumed to influence the interaction differently, such as for boys versus girls and for urban areas versus rural areas. Therefore, analyses were performed separately by sex and by residence area in two models. A more consistent method would be to group by sex separately in the separated analyses on rural versus urban living environment. However, the small cell size brought by increased levels of stratification (household wealth, parental education, sex, and residence area) was a major reason to not do so. Future studies with larger sample sizes may help to improve the interaction analysis between these various factors.

## 5. Conclusions

Father’s education level interacts with household wealth to influence obesity among girls and urban children in northeast China. With increase in household wealth, OR for the effect of father’s education on children’s obesity risk decreased. No interaction effect was found between household wealth and mother’s education.

## Figures and Tables

**Table 1:** Body mass index reference norm for screening obesity in Chinese children and adolescents [21].

Age (year)	Boys	Girls
9~	21.4	21.0
10~	22.5	22.1
11~	23.6	23.3
12~	24.7	24.5

**Table 2:** Waist–hip ratio reference norm for screening obesity in Chinese children and adolescents [22].

Age (year)	Boys	Girls
9~	0.88	0.92
10~	0.85	0.90
11~	0.83	0.88
12~	0.82	0.87

**Table 3:** Demographic characteristics of the 3670 children enrolled in the study.

Characteristic	All
Participants, N	3670
Age, years	10.8 ± 1.0
Sex, n (%)girls	1799 (49.0)
Residence area, n (%)urban	1645 (44.8)
Obesity defined by BMI, n (%)	623 (17.0)
Abdominal obesity defined by WHR, n (%)	297 (8.1)
Household wealth quintile	
Fifthquintile wealth (richest)	712 (19.4)
Fourthquintile wealth	584 (15.9)
Thirdquintile wealth	891 (24.3)
Second quintile wealth	757 (20.6)
First quintile wealth (poorest)	726 (19.8)
Parental education	
Father’s education, n (%)higher	1821 (49.6)
Mother’s education, n (%)higher	1802 (49.1)
Higher parental education in each household wealth quintile, n (%)	
Fifthquintile wealth (richest)	
Fathers with higher education	486 (68.3)
Mothers with higher education	494 (69.4)
Fourthquintile wealth	
Fathers with higher education	318 (54.5)
Mothers with higher education	330 (56.5)
Thirdquintile wealth	
Fathers with higher education	506 (56.8)
Mothers with higher education	491 (55.1)
Second quintile wealth	
Fathers with higher education	286 (37.8)
Mothers with higher education	287 (37.9)
First quintile wealth (poorest)	
Fathers with higher education	225 (31.0)
Mothers with higher education	200 (27.6)

**Table 4:** Prevalence of childhood obesity by subgroups.

	Obesity		Abdominal Obesity	
	*N* (%)	*p* ^1^	*N* (%)	*p* ^1^
Sex				
Boys	381 (20.4)	<0.001	147 (7.9)	0.57
Girls	242 (13.5)		150 (8.3)	
Residence area				
Urban	292 (17.8)	0.38	137 (8.3)	0.21
Rural	331 (16.4)		160 (7.9)	
Household wealth level				
Fifth quintile wealth	123 (17.3)	0.53	41 (5.8)	0.15
Fourth quintile wealth	111 (19.0)		54 (9.3)	
Third quintile wealth	148 (16.6)		67 (7.5)	
Second quintile wealth	126 (16.6)		66 (8.7)	
First quintile wealth	115 (15.8)		69 (9.5)	
Father’s education				
None/basic	317 (17.1)	0.74	154 (8.3)	0.71
Higher	306 (16.8)		143 (7.9)	
Mother’s education				
None/basic	300 (16.1)	0.22	161 (8.6)	0.37
Higher	323 (17.0)		136 (7.6)	

^1^ Chi-squared test was used.

**Table 5:** Interaction between parents’ education and household wealth quintiles on obesity and abdominal obesity risk.

	*n*	OR ^1^	95% CI	*p*-Value
Obesity				
Fathers with higher education		0.76	0.55, 1.04	0.08
Mothers with higher education		1.26	0.87, 1.81	0.20
Household wealth quintiles	3670	1.10	0.98, 1.23	0.10
Fathers with higher education × Household wealth quintiles		0.77	0.66, 0.91	<0.01
Mothers with higher education × Household wealth quintiles		1.12	0.94, 1.33	0.20
Constant		1.04	0.44, 2.47	0.92
Abdominal obesity				
Fathers with higher education		1.08	0.80, 1.44	0.59
Mothers with higher education		0.88	0.55, 1.40	0.56
Household wealth	3670	0.91	0.74, 1.13	0.38
Fathers with higher education × Household wealth quintiles		0.85	0.67, 1.08	0.17
Mothers with higher education × Household wealth quintiles		1.16	0.80, 1.66	0.41
Constant		0.02	0.00, 0.12	<0.01

^1^ Controlled for age (years), sex, residence area (urban/rural) and school (which school the children belonged to).

**Table 6:** Interaction between parent education and household wealth quintiles on obesity and abdominal obesity risk separated by sex.

	Model for Boys				Model for Girls			
	*n*	OR ^1^	95% CI	*p* Value	*n*	OR ^1^	95% CI	*p-*Value
Obesity								
Fathers with higher education		0.63	0.40, 1.00	0.02		0.93	0.60, 1.48	0.77
Mothers with higher education		1.36	0.94, 1.96	0.10		1.14	0.61, 2.14	0.66
Household wealth quintiles	1871	1.11	0.95, 1.30	0.17	1799	1.09	0.91, 1.31	0.30
Fathers with higher education × Household wealth quintiles		0.85	0.68, 1.07	0.15		0.69	0.51, 0.94	0.02
Mothers with higher education × Household wealth quintiles		1.10	0.83, 1.34	0.63		1.16	0.87, 1.54	0.28
Constant		1.36	0.27, 6.78	0.68		0.19	0.05, 0.77	0.02
Abdominal obesity								
Fathers with higher education		1.01	0.56, 1.81	0.98		1.15	0.71, 1.87	0.54
Mothers with higher education		1.02	0.56, 1.86	0.95		0.79	0.46, 1.34	0.35
Household wealth	1871	0.94	0.72, 1.24	0.65	1799	0.87	0.70, 1.09	0.22
Fathers with higher education × Household wealth quintiles		0.97	0.72, 1.31	0.85		0.79	0.51, 1.21	0.25
Mothers with higher education × Household wealth quintiles		1.01	0.67, 1.53	0.95		1.25	0.82, 1.91	0.28
Constant		0.02	0.00, 0.11	<0.01		0.04	0.00, 0.31	0.01

^1^ Controlled for age (years), residence area (urban/rural), and school (which school the children belonged to).

**Table 7:** Interaction between parent education and household wealth quintiles on obesity and abdominal obesity risk separated by residence area.

	Model for Urban				Model for Rural			
	*n*	OR ^1^	95% CI	*p-*Value	*n*	OR ^1^	95% CI	*p-*Value
Obesity								
Fathers with higher education		0.73	0.50, 1.07	0.10		0.75	0.48, 1.17	0.18
Mothers with higher education		1.35	0.87, 2.08	0.16		1.19	0.80, 1.77	0.35
Household wealth quintiles	1645	0.99	0.85, 1.15	0.85	2025	1.13	1.01, 1.26	0.04
Fathers with higher education × Household wealth quintiles		0.73	0.59, 0.90	0.01		0.93	0.72, 1.20	0.56
Mothers with higher education × Household wealth quintiles		1.24	0.89, 1.72	0.18		1.13	0.91, 1.42	0.25
Constant		0.58	0.18, 1.83	0.32		1.45	0.28, 7.56	0.64
Abdominal obesity								
Fathers with higher education		0.87	0.60, 1.26	0.43		1.37	0.77, 2.45	0.26
Mothers with higher education		0.98	0.66, 1.45	0.90		0.81	0.45, 1.45	0.45
Household wealth	1645	0.91	0.64, 1.29	0.58	2025	0.88	0.70, 1.10	0.23
Fathers with higher education × Household wealth quintiles		0.74	0.57, 0.96	0.03		1.07	0.70, 1.61	0.74
Mothers with higher education × Household wealth quintiles		1.26	0.87, 1.85	0.20		1.09	0.70, 1.68	0.68
Constant		0.02	0.00, 0.12	<0.01		0.01	0.00, 0.14	<0.01

^1^ Controlled by age (years), sex (boy/girl) and school (which school the children belonged to).

## Supplementary Materials

The following are available online at http://www.mdpi.com/1660-4601/15/8/1754/s1. Table S1: Interaction between parent education and household wealth categories on obesity and abdominal obesity risk, Table S2: Interaction between parent education and household wealth categories on obesity and abdominal obesity risk separated by sex, Table S3: Interaction between parent education and household wealth categories on obesity and abdominal obesity risk separated by residence area, Figure S1: OR (95%CI) for parent education level at different values of the household wealth categories among girls, Figure S2: OR (95%CI) for parent education level at different values of the household wealth categories among urban residences.
