# Supplementary material for: Interaction between Parental Education and Household Wealth on Children’s Obesity Risk
Source: Int J Environ Res Public Health. 2018 Aug 15;15(8):1754. doi: 10.3390/ijerph15081754 (PMC6121534; doi:10.3390/ijerph15081754)
Supplement: Supplementary file 1 [file ijerph-15-01754-s001.pdf]

Table S1. Interaction between parent education and household wealth categories on obesity and abdominal obesity risk.

	n	OR [1]	95% CI	*p*-Value
Obesity				
Fathers with higher education		0.69	0.48, 0.98	0.04
Mothers with higher education		1.33	0.91, 1.93	0.13
Household wealth quintiles	3670	1.17	0.93, 1.48	0.16
Fathers with higher education*Household wealth categories [2]		0.60	0.46, 0.79	0.001
Mothers with higher education*Household wealth categories [2]		1.25	0.94, 1.67	0.12
Constant		1.07	0.44, 2.58	0.88
Abdominal obesity				
Fathers with higher education		0.95	0.71, 1.27	0.71
Mothers with higher education		0.98	0.56, 1.71	0.93
Household wealth	3670	0.85	0.54, 1.34	0.47
Fathers with higher education*Household wealth categories [2]		0.62	0.45, 0.84	0.01
Mothers with higher education*Household wealth categories [2]		1.44	0.71, 2.91	0.29
Constant		0.02	0.00, 0.13	<0.001

[1] Controlled for age (in years), sex, residence area (urban/rural), and school (which school the children belonged to); [2] Categories classifying the lowest 40% wealth of households into “poor”, the highest 20% as “rich” and the rest as the “middle” group.

Table S2. Interaction between parent education and household wealth categories on obesity and abdominal obesity risk separated by sex.

	Model for boys				Model for girls			
	**n**	**OR** [1]	**95% CI**	***p*-Value**	**n**	**OR** [1]	**95% CI**	***p*-Value**
Obesity								
Fathers with higher education		0.60	0.38, 0.95	0.03		0.82	0.49, 1.37	0.42
Mothers with higher education		1.37	0.98, 1.93	0.06		1.25	0.63, 2.46	0.50
Household wealth quintiles	1871	1.24	0.91, 1.69	0.16	1799	1.11	0.75, 1.64	0.58
Fathers with higher education*Household wealth categories[2]		0.71	0.49, 1.04	0.07		0.50	0.29, 0.86	0.02
Mothers with higher education*Household wealth quintiles[2]		1.05	0.68, 1.62	0.83		1.50	0.92, 2.45	0.10
Constant		1.42	0.27, 7.49	0.66		0.20	0.05, 0.78	0.02
Abdominal obesity								
Fathers with higher education		0.96	0.54, 1.70	0.87		0.96	0.59, 1.54	0.85
Mothers with higher education		1.07	0.54, 2.11	0.83		0.87	0.44, 1.69	0.65
Household wealth	1871	0.89	0.51, 1.57	0.67	1799	0.84	0.53, 1.32	0.42
Fathers with higher education*Household wealth quintiles[2]		0.77	0.40, 1.48	0.41		0.55	0.26, 1.16	0.11
Mothers with higher education*Household wealth quintiles[2]		1.07	0.47, 2.42	0.87		1.74	0.81, 3.71	0.14
Constant		0.02	0.00, 0.09	<0.01		0.05	0.01, 0.31	0.31

[1] Controlled for age (in years), residence area (urban/rural), and school (which school the children belonged to); [2] Categories classifying the lowest 40% wealth of households into "poor", the highest 20% as "rich" and the rest as the "middle" group.

Table S3. Interaction between parent education and household wealth categories on obesity and abdominal obesity risk separated by residence area.

	Model for Urban				Model for Rural			
	n	OR [1]	95% CI	*p*-Value	*n*	OR [1]	95% CI	*p*-Value
Obesity								
Fathers with higher education		0.64	0.43, 0.95	0.03		0.73	0.44, 1.23	0.22
Mothers with higher education		1.46	0.96, 2.23	0.08		1.25	0.78, 2.00	0.32
Household wealth quintiles	1645	0.98	0.78, 1.23	0.83	2025	1.22	0.93, 1.59	0.14
Fathers with higher education*Household wealth categories [2]		0.51	0.36, 0.74	<0.01		0.87	0.50, 1.49	0.58
Mothers with higher education*Household wealth categories [2]		1.56	0.97, 2.51	0.06		1.25	0.78, 2.00	0.32
Constant		0.61	0.20, 1.89	0.36		1.34	0.25, 7.12	0.72
Abdominal obesity								
Fathers with higher education		0.77	0.50, 1.20	0.22		1.22	0.65, 2.28	0.50
Mothers with higher education		0.97	0.68, 1.40	0.87		0.95	0.44, 2.07	0.89
Household wealth	1645	0.96	0.53, 1.74	0.89	2025	0.78	0.50, 1.24	0.27
Fathers with higher education*Household wealth categories [2]		0.53	0.35, 0.82	0.01		0.80	0.36, 1.78	0.55
Mothers with higher education*Household wealth categories [2]		1.36	0.82, 2.26	0.21		1.54	0.50, 4.76	0.42
Constant		0.02	0.00, 0.12	<0.01		0.01	0.00, 0.15	<0.01

[1] Controlled by age (in years), sex (boy/girl), and school (which school the children belonged to). [2] Categories classifying the lowest 40% wealth of households into "poor", the highest 20% as "rich" and the rest as the "middle" group.

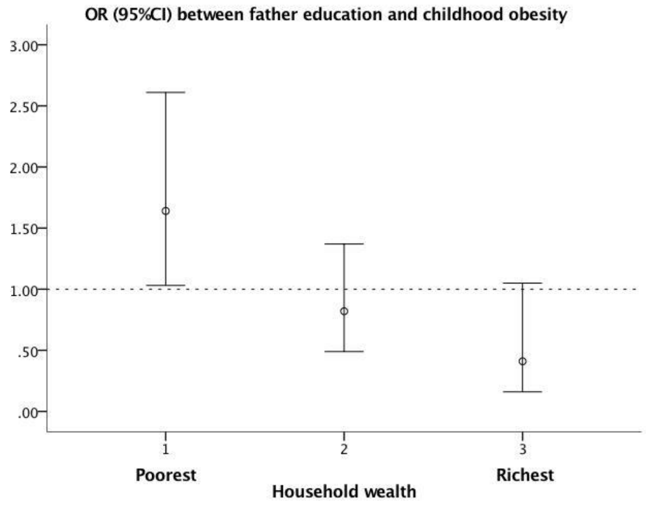

(A)

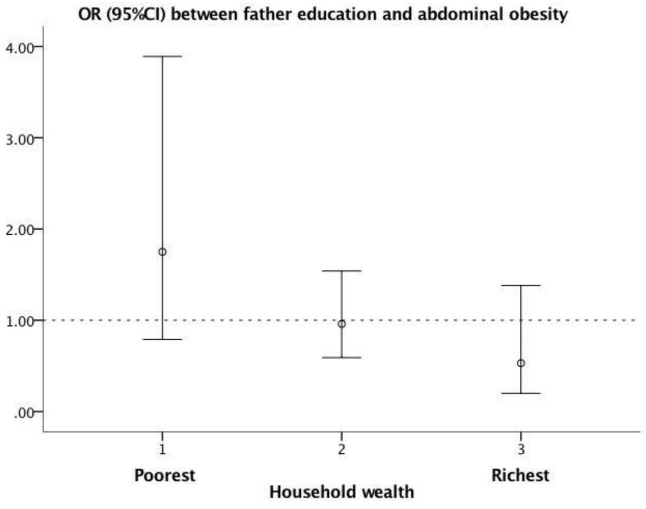

(B)

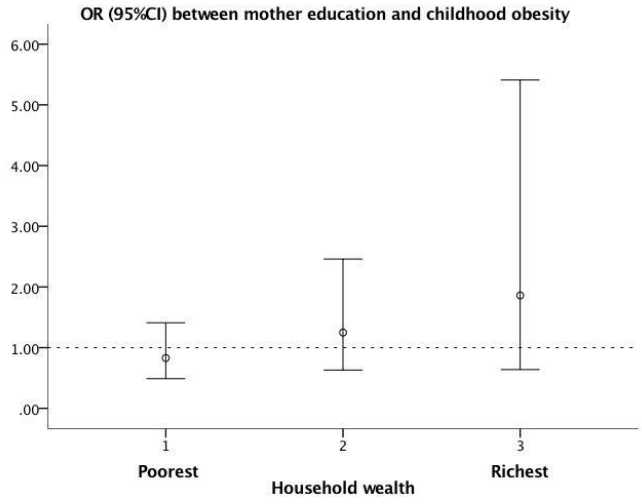

(C)

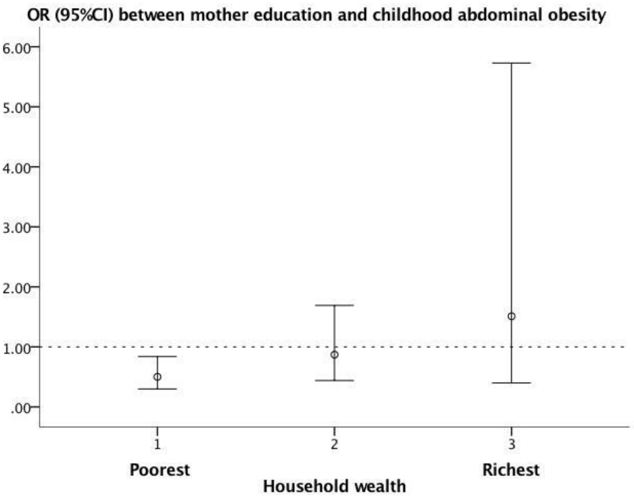

(D)

Figure S1. OR (95%CI) for parent education level at different values of the household wealth categories among girls. (**A**) OR (95% CI) between father education and obesity risk; (**B**) OR (95% CI) between father education and abdominal obesity risk; (**C**) OR (95% CI) between mother education and obesity risk; (**D**) OR (95% CI) between mother education and abdominal obesity risk.

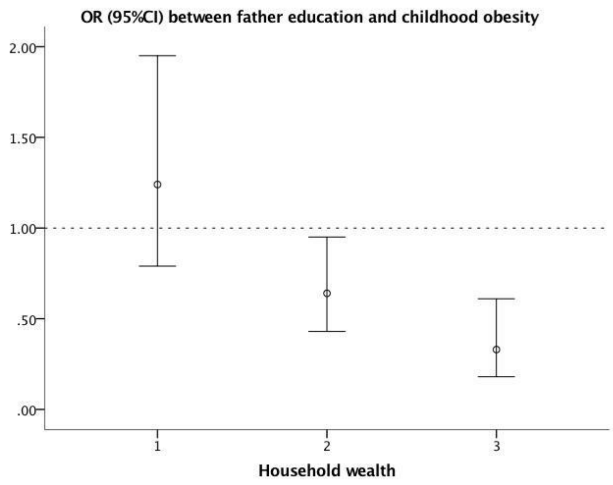

(A)

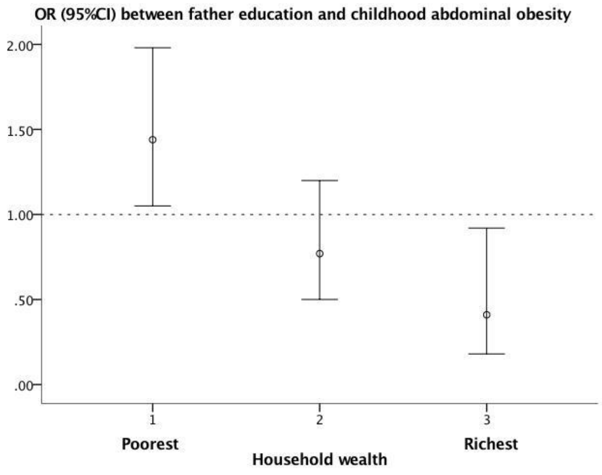

(B)

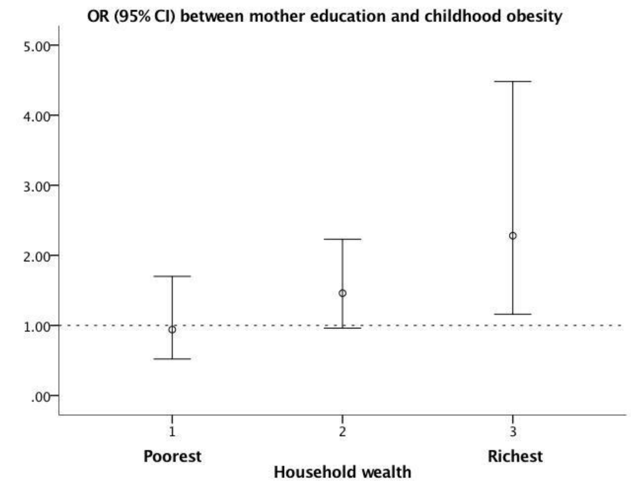

(C)

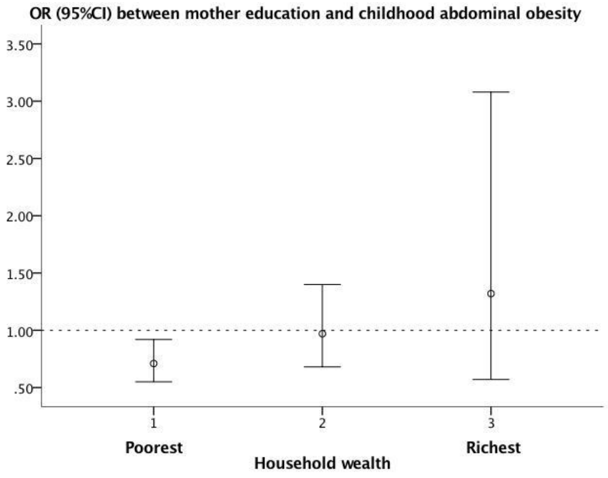

(D)

Figure S2. OR (95%CI) for parent education level at different values of the household wealth categories among urban residences. (**A**) OR (95% CI) between father education and obesity risk; (**B**) OR (95% CI) between father education and abdominal obesity risk; (**C**) OR (95% CI) between mother education and obesity risk; (**D**) OR (95% CI) between mother education and abdominal obesity risk.
